# Differences in the expression of DNA methyltransferases and demethylases in leukocytes and the severity of pulmonary arterial hypertension between ethnic groups

**DOI:** 10.14814/phy2.15282

**Published:** 2022-05-17

**Authors:** Catherine A. D’Addario, Gregg M. Lanier, Christina Jacob, Natalie Bauer, Jenny L. Hewes, Aritra Bhadra, Sachin A. Gupte

**Affiliations:** ^1^ Department of Pharmacology New York Medical College Valhalla NY USA; ^2^ Department of Cardiology, and Heart and Vascular Institute Westchester Medical Center and New York Medical College Valhalla NY USA; ^3^ Department of Pharmacology College of Medicine University of South Alabama Mobile AL USA

**Keywords:** epigenetics, humans, pulmonary hypertension

## Abstract

The loss of ten‐eleven translocation (TET2) methylcytosine dioxygenase expression contributes to the pathobiology of pulmonary arterial hypertension (PAH). However, whether the expression and activity of other TETs and DNA methyltransferases (DNMTs) are altered in PAH remains enigmatic. Therefore, our objective was to determine the expression of DNMT (1, 3a, and 3b) and TET (1, 2, and 3) and their total activity. We assessed the expression of DNMT and TET enzymes in the leukocytes and their activity in extracellular vesicles (EVs). Expression of *DNMT (1*, *3a*, *and 3b)*, *TET (2* and *3)* in leukocytes, and total activity in EVs, from PAH patients was higher than in healthy controls. Additionally, we noticed there were difference in expression of these epigenetic enzyme based on ethnicity and found higher *DNMT1* and lower *TET2*/*TET3* expression in Caucasian than Hispanic/African American (combine) patients. Since loss‐of‐function mutation(s) and down‐regulation of TET enzymes are associated with hematological malignancies and cytokine production, we determined the expression of genes that encode cytokines in samples of Caucasian and Hispanic/African American patients. Expression of *IL6*, *CSF2*, and *CCL5* genes were higher in the leukocytes of Caucasian than Hispanic/African American patients, and *CSF2* and *CCL5* negatively correlated with the decreased expression of *TET3*. Interestingly, the expression of gene encoding CD34, a marker of myeloid and lymphoid precursor cells, and CD163, a monocyte/macrophage protein, was higher in the leukocytes of Caucasian than Hispanic/African American patients. Furthermore, Hispanic/African American patients having higher *TET2*/*TET3* expression had higher pulmonary capillary wedge pressure. In conclusion, our results revealed higher *DNMT1* and lower *TET2*/*TET3* in Caucasian than Hispanic/African American patients together potentially augmented genes encoding inflammation causing cytokines, and CD34^+^‐derived immunogenic cells, and the severity of PAH.

## Graphical abstract

This study revealed higher DNMT1 and lower TET2/TET3 in Caucasian than Hispanic/African American patients together potentially augmented genes encoding inflammation causing cytokines, and CD34+‐derived immunogenic cells, and the severity of PAH.

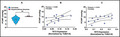

## 1 INTRODUCTION

Evidence that alteration of DNA and histone methylation or acetylation (epigenetic modifications) are associated with the development of all types of pulmonary hypertension is accumulating (Napoli et al., 2019). It has been proposed, for example, that altered methylation and acetylation marks on histones produce a persistent inflammatory phenotype in fibroblasts and an apoptosis‐resistant and hyperproliferative phenotype of endothelial and smooth muscle cells from hypertensive patients and animal models (Hu et al., 2019). DNA methylation is regulated by DNA methyltransferases (DNMTs; 1, 3a, and 3b) and TET methylcytosine dioxygenases (TETs; 1, 2, and 3), DNA demethylases (Lachat et al., 2020).

In recent studies, we and others observed that differential DNA methylation and a reduction in TET2 mRNA expression may be key to the pathogenesis of pulmonary hypertension in mice and humans (Joshi et al., 2020; Potus et al., 2020). In those studies, our laboratory found that DNA methylation‐dependent transcription of pulmonary hypertension‐related genes and *Tet2* expression is suppressed in the lungs of hypoxic mice (Joshi et al., 2020). Moreover, we observed that hypoxia‐induced metabolic reprogramming contributes to the reduction in *Tet2* expression, and that inhibiting glucose‐6‐phosphate dehydrogenase activity increases pulmonary *Tet2* expression in hypoxic mice and reduces pulmonary hypertension (Joshi et al., 2020). Others have found that inherited and acquired *TET2* abnormalities occur in only 0.39% of pulmonary arterial hypertension (PAH) cases, but lower expression of *TET2* in mononuclear cells was detected, with elevated pro‐inflammatory cytokines, in >86% of inflammation‐associated PAH cases (Potus et al., 2020). Consistent with those findings, *Tet2* deletion leads to increases in right ventricular (RV) pressure in 7‐month‐old, but not in 2‐month‐old, mice (Potus et al., 2020). Altogether these findings highlight an association between reduced *TET2* and the pathogenesis of pulmonary hypertension in humans and mice.

While loss‐of‐function mutations in *TET2* are associated with diverse blood cell malignancies in humans (Solary et al., 2014), loss of a single *TET* enzyme is not sufficient to efficiently promote malignancy and promote synthesis of pro‐inflammatory cytokines (An et al., 2015). Although reduction of TET2 has been implicated as a contributing factor in the development of PAH, information regarding the expression of DNMT and other TET enzymes is differentially regulated in patients with PAH remains unclear. More so, the activity of these epigenetic writers and eraser enzymes, which are metabolically regulated, remains undetermined in PAH cases. Therefore, we assessed *DNMT* (1, 3a, and 3b) and *TET* (1, 2, and 3) expression in leukocytes as well as their activity in extracellular vesicles (EVs), submicron intact vesicles (Blair et al., 2016), isolated from plasma collected from scleroderma (scl) and idiopathic (i) PAH patients. Furthermore, whether differential expression of DNMTs or TETs influences maladaptive cytokine gene expression and severity of PAH is unknown. Therefore, we analyzed the expression of DNMTs and TETs enzyme in Caucasian, Hispanic, and African American patients with PAH. Our findings suggest that *DNMT (1*, *3a*, and *3b*), *TET2*, and *TET3* are increased in humans with PAH. What is more, the results suggest that higher *DNMT1* and lower *TET2* and *TET3* expression is directly correlated with increased cytokines (*IL6* and *CCL5*) and severity of PAH in Caucasian and Hispanic/African American (combined) patients.

## 2 MATERIALS AND METHODS

### 2.1 Drugs and reagents

All chemicals and reagents were purchased from Sigma, Thermo‐Fisher, R&D Systems, MyBioSource, and VWR.

### 2.2 Collection of blood from control individuals and PAH patients

All protocols were approved by the New York Medical College and Westchester Medical Center Institutional Review Board (Protocol #11775). Patient demographics are presented in Table 1. Blood samples were collected into heparinized tubes from PAH patients and control individuals by venipuncture and stored at 4 °C for 24 h. White blood cells (WBCs/leukocytes) were isolated as described previously (Hashimoto et al., 2020). Briefly, the cells were collected by centrifugation at 300 g for 5 min at 4 °C. The supernatant was aspirated, and the cell pellet was resuspended in 10 ml of red cell lysis buffer (0.15 M NH_4_Cl; 0.01 M KHCO_3_; 0.1 M Na_2_EDTA, pH 7.2–7.4) and incubated on ice for 15 min. The cells were then collected by centrifugation (1200 *g*), washed twice with PBS and immediately suspended in Qiazol (700 μl; Qiagen), after which total RNA was isolated and used for real‐time PCR as described below. We used leukocytes for gene expression analysis, as a surrogate for lung tissue or pulmonary artery, because they are increased in blood of PAH patients and a previous study has measured TET expression in mononuclear blood cells from PAH patients (Potus et al., 2020).

**TABLE 1:** Demographics and treatment history of PAH cases

Subject ID	Date of sample collection	Subject age/sex	Ethnicity	Treatment
Scleroderma‐associated PAH				
A2^a^	10/25/17	56/F	Caucasian	SQ remodulin, riociguat 2.5 TID
A3^a^	10/31/17	59/F	Hispanic	IV remodulin 40, ambrisentan 10 mg daily, tadalafil 40 mg daily
A4^a^	11/01/17	60/F	Hispanic	tadalafil 40 mg daily, macitentan 10 mg daily, selexipag 400 μg BID
A5^a^	12/05/17	75/F	Caucasian	IV remodulin 20, riociguat 2.5 mg TID, ambrisentan 10 mg daily
B	04/30/18	78/F	Caucasian	IV remodulin 112, macitentan 10 mg dialy, tadalafil 40 daily
C	05/01/18	70/F	Caucasian	ambrisentan 10 mg daily, tadalafil 40 mg daily
F	09/12/18	81/F	Hispanic	selexipag 800 μg BID, sildenafil 40 mg TID, macitentan 10 mg daily
I	12/3/18	53/F	Hispanic	none at time of enrollment
Idiopathic PAH				
B1^a^	11/01/17	54/F	Caucasian	IV remodulin 72, riociguat 2.5 mg TID, macitentan 10 mg daily
B2	010/9/18	35/F	African American	macitentan 10 mg daily, riociguat 2.5 mg TID, treprostinil 60
J	03/19/19	71/F	Caucasian	riociguat 2.5 mg TID, macitentan 10 daily
M	10/07/19	71/F	Caucasian	treprostinil IV 69 ng/kg/min, macitentan 10 mg daily, sildenafil 20
O	05/27/20	61/F	Caucasian	tadalafil 40 mg daily
Controls without PAH				
C1	08/17/17	39/M	Caucasian	N/A
C2	08/17/17	32/F	Caucasian	N/A
C3	12/05/17	24/F	Caucasian	N/A
C4	12/12/17	64/F	Caucasian	N/A
C5	12/12/17	36/F	Indian	N/A
C6	12/12/17	28/F	Caucasian	N/A
G	09/12/18	32/F	Caucasian	N/A

Abbreviations: PAH, pulmonary arterial hypertension; N/A, not applicable.

^a^ Results of these patients were from a recent publication (REF #14).

### 2.3 Quantitative real‐time PCR

Real time PCR was used to analyze mRNA expression. Briefly, total RNA was extracted from lungs and human leukocytes using a Qiagen miRNeasy kit (Cat # 217004). The quality and concentration of the input RNA were measured on the Synergy HT Take3 Microplate Reader (BioTek) and cDNA was prepared using SuperScript™ IV VILO™ Master Mix with ezDNase™ Enzyme (Cat # 11766500, Invitrogen) for mRNA. Quantitative PCR was performed in duplicate using TaqMan™ Fast Advanced Master Mix (Cat # 44‐445‐57) for mRNA using a Mx3000p Real‐Time PCR System (Stratagene). The primers for the qPCR were purchased from Thermo Fisher Scientific/TaqMan. mRNA levels were normalized to internal control *TUBA1A*, and relative mRNA expression was reported.

### 2.4 Isolation and identification of EV

Because we used the leukocytes to extract RNA for PCR, we could measure activity of epigenetic enzymes in the immune cells. Therefore, we isolated EVs from plasma collected from PAH patients and healthy controls as previously described (Blair et al., 2016). Briefly, we generated platelet‐rich plasma by centrifugation of samples at 1500 × *g* for 20 min at room temperature. The platelet‐rich plasma was then used to generate platelet‐free plasma (PFP) by centrifugation at 13,000 *g* for 10 min at room temperature. PFP was diluted 1:40 with PBS and ultracentrifuged for 60 min at 100,000 *g* at 4° in an Optima XE‐100 Ultracentrifuge (Beckman Coulter). The supernatant plasma was then removed, and the recovered EV pellet was dissociated in PBS. We quantitated EVs using Zeta potential analysis and identified by transmission electron microscopy (TEM).

### 2.5 Measurement of DNMT and TET activity

DNMT and TET activity was measured using kits from Epigentek. DNMT and TET activity were measured in EVs isolated from plasma collected from PAH patients and healthy controls.

### 2.6 Isolation of leukocytes and flow cytometry

Blood samples were collected from PAH patients. After red blood cell lysis using lysing buffer (BD Biosciences), 10^6^ cells suspended in 90 µl of buffer were treated with 10 µl of FcR blocking reagent (Miltenyi Biotec) for 10 min at 4 °C and stained with 10 µl of fluorescent antibodies for 15 min at 4 °C. We used fluorescein (FITC)‐conjugated anti‐CD34 antibody purchased from Miltenyi Biotec. Flow cytometry was performed as described before (Hashimoto et al., 2020). Negative control (without) primary antibody treated cells were used each time as validation of antibodies.

### 2.7 Statistical analysis

Statistical analysis was performed using GraphPad Prism 9 software. Values are presented as mean ± standard error (SE). Statistical comparisons of samples were made for two groups with Mann–Whitney test. Values of *p* < 0.05 were considered significant.

## 3 RESULTS

### 3.1 Treatment and hemodynamic changes in scleroderma‐associated and idiopathic PAH cases

We collected blood from eight scl‐PAH and five i‐PAH patients and seven healthy controls. We had four Caucasians and four Hispanics in the scl‐PAH group, four Caucasians and one African American in the i‐PAH group, and six Caucasians and one Indian American in the control group. Their ages ranged from 56 to 78 years in the PAH group and from 24 to 64 years in the control group (Table 1). Right atrial pressure (RAP), pulmonary arterial pressure (PAP), pulmonary capillary wedge pressure (PCWP), transpulmonary gradient (TPG), pulmonary vascular resistance (PVR), and cardiac output and index did not differ between the scl‐PAH and i‐PAH groups (Table 2).

**TABLE 2:** Hemodynamic results of scleroderma‐associated and idiopathic PAH cases

ID	RAP	SPAP	DPAP	MPAP	PCWP		TPG		PVR	Fick CO	Fick CI
Scleroderma‐associated PAH											
A2^a^	12	88	33	56	9	47		7.66		8.63	4.1
A3^a^	8	55	13	34	11	23		6.68		3.44	1.9
A4^a^	5	60	21	36	11	25		7.39		3.38	2.3
A5^a^	2	86	28	48	2	49		17.56		2.79	1.7
B	1	58	13	31	4	27		5.7		4.73	3
C	4	55	15	26	8	18		4.04		4.45	2.7
F	12	95	35	59	11	48		17.91		2.68	1.87
I	15	75	30	48	12	36		7.7		5.11	2.9
Mean	8.1	70.0	23.2	41.8	9.9	32.2		8.6		4.7	2.6
SD	5.3	16.1	8.6	11.5	5.4	13.0		5.4		2.0	0.8
Idiopathic PAH											
B1^a^	3	76	24	44	6	38		8.59		4.42	2.5
B2	6	53	21	33	11	23		3.82		3.88	2.2
J	15	86	27	45	6	39		12.18		2.1	1.2
M	6	91	33	56	9	47		8.27		5.68	2.6
O	13	83	35	51	11	40		8.23		4.86	2.3
Mean	8.6	77.8	28.0	45.8	8.6	37.4		8.2		4.2	2.2
SD	5.1	14.9	5.9	8.6	2.5	8.8		3.0		1.3	0.6

Abbreviations: CI, Fick cardiac index (L/min/m^2^); DPAP, diastolic pulmonary artery pressure (mmHg); Fick CO, fick cardiac output (liters/min); MPAP, mean pulmonary artery pressure (mmHg); PCWP, mean pulmonary capillary wedge pressure (mmHg); PVR, pulmonary vascular resistance (Woods units); RAP, mean right atrial pressure (mmHg); SD, standard deviation; SPAP, systolic pulmonary artery pressure (mmHg); TPG, transpulmonary gradient.

^a^ Results of these patients were from a recent publication (REF #14).

### 3.2 DNMT and TET expression and activity in leukocytes and EV from PAH patients

**FIGURE 1:**
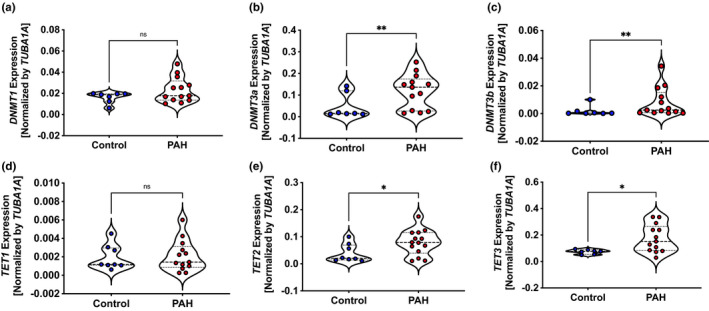
Expression of DNMTs and TETs elevated in leukocytes from scleroderma‐associated and idiopathic‐PAH patients than their controls. Using RT‐PCR, *DNMT*, and *TET* expression were determined in leukocytes (all white blood cells) isolated from peripheral blood collected from scleroderma‐associated and idiopathic (scl/‐i) PAH patients and their healthy controls. (a–c) Violin plot shows expression of *DNMTs* increased in leukocytes (all white blood cells) from scl/i‐PAH patients than their healthy controls. (d–f) Expression of *TET1* did not increase, but *TET2* and *TET3* was elevated in leukocytes of scl/i‐PAH patients than their healthy controls. *N* = 7 in the control group and *N* = 13 patients in the scl/i = PH group. Comparisons were made using Student's *t*‐test with Welch's correction. *p < 0.05 and ** *p* < 0.005

**FIGURE 2:**
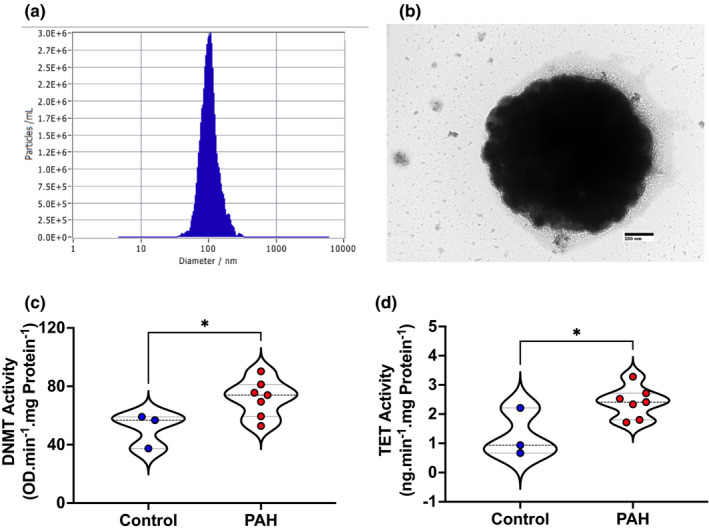
Total DNMT and TET activity elevated in extracellular vesicles from scleroderma‐associated and idiopathic‐PAH patients than their controls. (a, b) Extracellular vesicles were isolated from the plasma of scl/i‐PAH patients and their controls and confirmed with zeta potential analysis and transmission electron microscopy. DNMT and TET activity was determined in EV isolated from plasma of 2–3 control individuals and 2–3 scl/i‐PAH patients pooled together. (c, d) DNMT and TET activity was higher in EV isolated from plasma scl/I‐PAH patients than the healthy control individuals. DNMT and TET activity was measured in *N* = 3 for controls and *N* = 7 for PAH patients pooled plasma. Comparisons were made using Student's *t*‐test with Welch's correction. *p < 0.05

To determine whether the expression and activity of DNMTs were modified in PAH, we assessed expression of three major *DNMTs* (1, 3a, and 3b) in leukocytes and total DNMT activity in EVs from PAH patients and controls. Real‐time PCR showed that *DNMT3a* and *DNMT3b*, but not *DNMT1*, expression was significantly higher in the PAH than the control group (*p* < 0.05; Figure 1a–c). Similarly, we measured the expression of TET enzymes. While expression of *TET1* did not change, *TET2* and *TET3* increased in the PAH than the control group (*p* < 0.05; Figure 1d–f). We did not find a correlation between age and expression of DNMTs and TETs (data not shown). To determine whether there was a corresponding increase in DNMT and TET activity in PAH cases, we isolated EVs from PAH patients and controls. After using zeta potential analysis and electron microscopy to confirm the diameter and appearance of the isolated EV (Figure 2a,b), we determined their associated enzyme activity. Total activity of DNMT and TET was significantly higher in EVs from the PAH patients (Figure 2c,d).

### 3.3 DNMT and TET expression in Caucasian vs Hispanic/African American (combine) PAH patients

**FIGURE 3:**
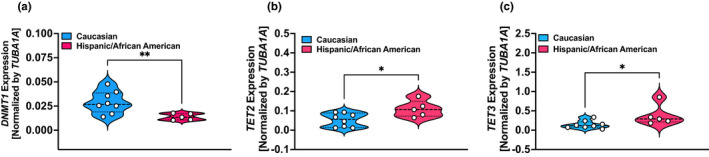
Expression of *DNMTs* and *TETs* in leukocytes from Caucasian than Hispanic/African American patients with scleroderma‐associated and idiopathic‐PAH patients. Expression of *DNMT* and *TET* was compared between the different ethnic groups with scl/i‐PAH. (a) *DNMT1* expression was higher and (b, c) *TET2*/*TET3* was lower in leukocytes from Caucasian than Hispanic/African American scl/i‐PAH patients. *N* = 8 in the Caucasian group and *N* = 5 in the Hispanic (*N* = 4)/African American (*N* = 1) group. Comparisons were made using Student's *t*‐test with Welch's correction. *p < 0.05 and **p < 0.005

Since lower expression of *TET2* in mononuclear cells was detected in >86% of inflammation‐associated PAH cases (Potus et al., 2020), our findings suggest higher *TET2* expression in PAH patients with scleroderma was somewhat perplexing and surprising to us. Therefore, to determine the potential cause for the differences in the results of the present and previous studies, we analyzed/compared the expression of DNMTs and TETs between different ethnic groups. Furthermore, along those lines, we noticed the difference in their expression based on the ethnicity and hence separated the patients based on ethnicity and in Caucasian and non‐Caucasian groups to determine differences in the expression and activity of epigenetic enzymes and cytokines. In the non‐Caucasian group we combined Hispanics and African American patients. Interestingly, we found higher *DNMT1* and lower *TET2* and *TET3* expression in leukocytes of Caucasians as compared with Hispanic/African American (combined) patients (Figure 3a–c), and this was not because of differences in the number of leukocytes between Caucasian and Hispanic/African American patients (Table 3).

**TABLE 3:** CBC of Caucasian and Hispanic/African American PAH cases

	Caucasians	Hispanics and African American
WBC (K/mm^3^)	6.9 ± 2.0	5.4 ± 1.7
Neutrophils (%)	71.0 ± 10.0	65.4 ± 10.7
Lymphocytes (%)	18.3 ± 9.4	22.3 ± 11.1
Monocytes (%)	6.8 ± 1.4	8.4 ± 2.6
RBC (10^12^/L)	4.1 ± 0.9	4.0 ± 0.5
Hgb (mmol/L)	10.9 ± 2.8	11.4 ± 0.5
Platelet (K/μl)	200.6 ± 81.4	194.8 ± 22.4

Note

Mean ± SD.

### 3.4 Cytokine levels in Caucasian versus Hispanic/African American (combine) PAH patients

**FIGURE 4:**
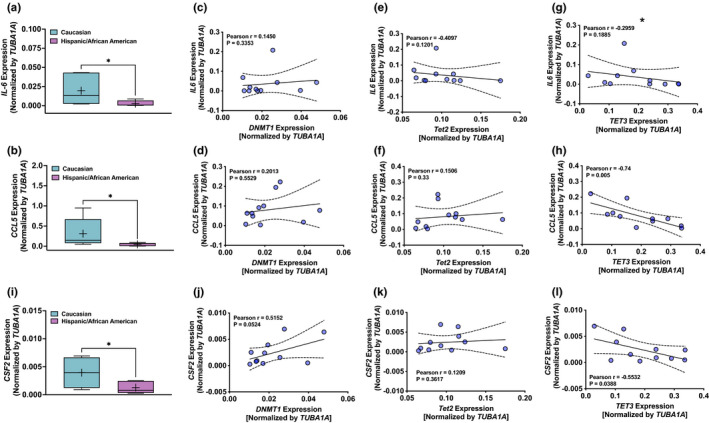
Expression of cytokines in leukocytes from Caucasian than Hispanic/African American patients with scleroderma‐associated and idiopathic‐PAH patients. Expression of genes encoding cytokines was compared between the different ethnic groups with scl/i‐PAH. (a, b) *IL*‐*6* and *CCL5* expression was higher in leukocytes from Caucasian as compared with Hispanic/African American patients. Pearson's correlation between (c, d) *DNMT1*, (e, f) *TET2* and (g, h) *TET3*, and cytokines in leukocytes from Caucasian than Hispanic/African American scl/i‐PAH patients is shown. (i) *CSF2* expression was higher in leukocytes from Caucasian as compared with Hispanic/African American patients. Pearson's correlation between (j) *DNMT1*, (k) *TET2* and (l) *TET3*, and *CSF2* expression in leukocytes from Caucasian than Hispanic/African American scl/i‐PAH patients is shown. A significant negative correlation between decreased *TET3* and *CSF2* was observed. *N* = 6 in the Caucasian group and *N* = 5 in the Hispanic (*N* = 4)/African American (*N* = 1) group. Comparisons were made using Student's *t*‐test with Welch's correction. *p < 0.05 and **p < 0.005

Altered DNA methylation and reduced expression of TET enzymes have been implicated to elevate inflammatory cytokines and associated with hematological disorders or malignancies (An et al., 2015; Solary et al., 2014). Furthermore, increased cytokine levels have been concurrently observed with the loss‐of‐function mutation in *TET2* and reduction of *TET2* expression in PAH patients (Potus et al., 2020). Therefore, we determined expression of *IL6*, *CCL5*, *CCL2*, and *CSF2* genes in leukocytes of Caucasian and Hispanic/African American patients. We found Caucasian as compared with Hispanic/African American patients expressed more *IL6* and *CCL5* (Figure 4a,b). Next, to determine if elevation of these cytokines are dependent on altered *DNMT1* and/or *TET2*/*TET3* expression, we performed Pearson correlation analysis. Interestingly, while neither *IL6* nor *CCL5* depend on DNMT1 (Figure 4c,d) and TET2 (Figure 4e,f), expression of both the cytokine genes and especially *CCL5* gene negatively correlated (*p* < 0.05) with *TET3* expression (Figure 4g,h). Similarly, we found *CSF2* expression was higher in Caucasian versus Hispanic/African American patients and *CSF2* expression negatively correlated (*p* < 0.05) with *TET3* expression (Figure 4i–l). *CCL2* expression did not differ between Caucasian and Hispanic/African American patients.

### 3.5 *CD163* gene levels positively correlates with the *DNMT1* expression in Caucasian versus Hispanic/African American (combine) PAH patients

**FIGURE 5:**
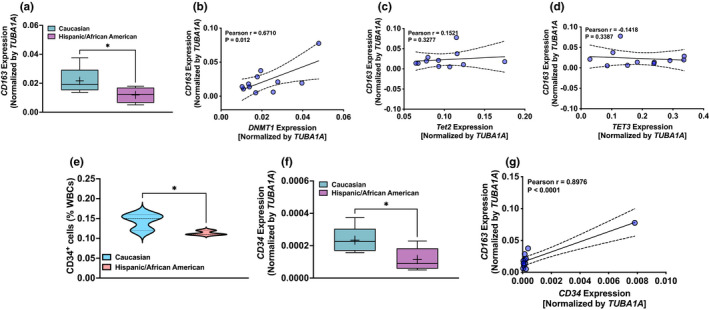
Expression of *CD163* and *CD34* in leukocytes from Caucasian and Hispanic/African American patients with scleroderma‐associated and idiopathic‐PAH patients. Expression of genes encoding *CD163*, a marker of monocytes/macrophages, and *CD34*, a marker of hematopoietic stem cells, was compared between the different ethnic groups with scl/i‐PAH. (a) *CD163* expression was higher in leukocytes from Caucasian as compared with Hispanic/African American patients. Pearson's correlation between (b) *DNMT1*, (c) *TET2* and (d) *TET3*, and *CD163* expression show a significant positive correlation between *DNMT1* and *CD163*. (e, f) Flow cytometry results demonstrating more CD34^+^ cells in blood and higher expression *CD34* in leukocytes from Caucasian as compared with Hispanic/African American patients. (g) Pearson's correlation between *CD34* and *CD163* is demonstrated. *N* = 6 in the Caucasian group and *N* = 5 in the Hispanic (*N* = 4)/African American (*N* = 1) group. Comparisons were made using Student's *t*‐test with Welch's correction. *p < 0.05 and **p < 0.005

Next, we determined expression of genes encoding immunogenic cell markers and found higher expression of *CD163* gene in leukocyte samples of Caucasian as compared with Hispanic/American African patients (Figure 5a). Furthermore, expression of *CD163* depended on DNMT1 (Figure 5b) but not on TET2/TET3 (Figure 5c,d). Additionally, although the expression of *ITGAM*, a gene that encodes integrin αM chain expressed on monocytes and macrophages, trended to be higher in Caucasian (0.10 ± 0.03) than Hispanic/African American (0.06 ± 0.01) patients, we did not find significant differences between the groups and correlation with the expression of DNMT1.

### 3.6 CD34^+^ cells and *CD34* gene levels in Caucasian versus Hispanic/African American (combine) PAH patients

CD34^+^ cells are precursors of immunogenic myeloid and lymphoid cells. These cells are increased in blood and lungs of PAH patients. Therefore, we determined whether these cells and the gene encoding CD34 protein are elevated in Caucasian compared to Hispanic/African American patients with PAH. As we expected, CD34^+^ cell numbers and the expression of the gene encoding *CD34* were higher in Caucasian than Hispanic/African American patients (Figure 5e,f) Furthermore, we found expression of *CD34* and *CD163* positively correlated (Figure 5g).

### 3.7 Correlation between epigenetic enzymes and PCWP in Caucasian versus Hispanic/African American (combine) PAH patients

**FIGURE 6:**
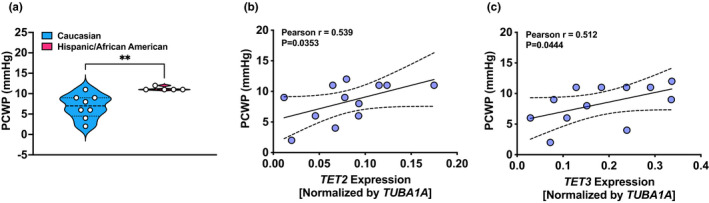
Severity of PAH correlates with lower *TET2* and *TET3* expression in Caucasian and Hispanic/African American patients with scleroderma‐associated and idiopathic‐PAH patients. (a) Pulmonary capillary wedge pressure (PCWP) was lower in Caucasian than Hispanic/African American scl/i‐PH patients. Pearson's correlation analysis demonstrates a positive correlation between (b) *TET2* and (c) *TET3*, and PCWP. *N* = 8 in the Caucasian group and *N* = 5 in the Hispanic (*N* = 4)/African American (*N* = 1) group. Comparisons were made using Student's *t*‐test

To determine whether there is any difference in the severity of PAH between Caucasian and Hispanic/African American patients, we compared the hemodynamic results. Our results revealed lower (*p* = 0.004) PCWP (Figure 6a) and a trend toward higher PVR (Caucasian: 9.74 ± 1.49 vs. Hispanic/African American: 6.40 ± 0.89; in mmHg; *p* = 0.075; Table 4) in Caucasian than Hispanic/African American patients. Next, to determine whether the expression of *DNMT1* and/or *TET2*/*TET3* has any bearing on the severity of PAH in Caucasian and Hispanic/African American patients, we performed Pearson correlation analysis. We found PCWP positively correlated (*p* < 0.05) with the expression of *TET2* and *TET3* (Figure 6b,c) and not with *DNMT1* (data not shown).

**TABLE 4:** Hemodynamic results of Caucasian and Hispanic/African American PAH cases

ID	RAP	SPAP	DPAP	MPAP	PCWP	TPG	PVR	Fick CO	Fick CI
Caucasian									
A2^a^	12	88	33	56	9	47	7.66	8.63	4.1
A5^a^	2	86	28	48	2	49	17.56	2.79	1.7
B	1	58	13	31	4	27	5.7	4.73	3
C	4	55	15	26	8	18	4.04	4.45	2.7
B1^a^	3	76	24	44	6	38	8.59	4.42	2.5
J	15	86	27	45	6	39	12.18	2.1	1.2
M	6	91	33	56	9	47	8.27	5.68	2.6
O	13	83	35	51	11	40	8.23	4.86	2.3
Mean	7.0	77.9	26.0	44.6	6.9	38.1	9.0	4.7	2.5
SD	5.5	13.9	8.3	11.0	2.9	10.8	4.2	2.0	0.9
Hispanic and African American									
A3^a^	8	55	13	34	11	23	6.68	3.44	1.9
A4^a^	5	60	21	36	11	25	7.39	3.38	2.3
B2	6	53	21	33	11	23	3.82	3.88	2.2
F	12	95	35	59	11	48	17.91	2.68	1.87
I	15	75	30	48	12	36	7.7	5.11	2.9
Mean	9.2	67.6	24.0	42.0	11.2^b^	31.0	8.7	3.7	2.2
SD	4.2	17.6	8.6	11.3	0.4	10.9	5.4	0.9	0.4

Abbreviations: CI, Fick cardiac index (L/min/m^2^); DPAP, Diastolic pulmonary artery pressure (mmHg); Fick CO, Fick cardiac output (liters/min); MPAP, Mean pulmonary artery pressure (mmHg); PCWP, Mean Pulmonary capillary wedge pressure (mmHg); PVR, Pulmonary vascular resistance (Woods units); RAP, Mean right atrial pressure (mmHg); SD, standard deviation; SPAP, Systolic pulmonary artery pressure (mmHg); TPG, Transpulmonary gradient.

^a^ Results of these patients were from a recent publication (REF #14).

^b^ *p* < 0.005 versus Caucasians.

## 4 DISCUSSION

PAH is a heterogeneaous and complex angio‐proliferative disease. PAH also exhibits cancer‐like physiognomies, and hence some studies have characterized PAH as cancer‐like disease (Culley & Chan, 2018; Leopold & Maron, 2016). One of the hallmarks of cancer is an alteration of DNA and histone methylation resulting in increased expression of oncogenes and/or decreased expression of tumor suppressor genes (Ehrlich, 2019; Samudio‐Ruiz & Hudson, 2012). Increased methylation frequently silences DNA repair genes and miRs involved in cancer biology (Lakshminarasimhan & Liang, 2016). As in cancer, differential DNA and histone methylation is associated with different sub‐types of PAH in experimental models and humans (Archer et al., 2010; Joshi et al., 2020; Ke et al., 2018; Napoli et al., 2019; Potus et al., 2015, 2020). Because methyltransferases and demethylases regulate the level of DNA and histone methylation and can thus alter gene transcription (Lachat et al., 2020), we investigated expression and activity of DNMT and TET enzymes in leukocytes and EVs from PAH patients.

While *DNMT1*, *DNMT3a*, and *DNMT3b* expression increased in leukocytes from PAH patients as compared to healthy controls, our data showing higher *DNMT1* expression and PCWP in Caucasian than Hispanic/African American PAH patients suggests Caucasian patients with higher *DNMT1* appear to have more severe PAH. Therefore, this implies augmented expression and activity of DNMTs, at least partly, contributed to the development and progression of PAH. In that regard, a recent study suggests expression of *DNMT3b* increases and other *DNMT*s decrease in lungs of congestive heart disease‐associated PAH, and knockdown of *Dnmt3b* in rats augments monocrotaline and hypoxia induced pulmonary hypertension (Yan et al., 2020). Another recent study shows an association between up‐regulation of *DNMT1* and down‐regulation of *BMPR2* expression in lungs of PAH patients (Bisserier et al., 2021). Similarly, increased DNA methylation in the lungs of hypoxic mice reduces expression of genes encoding proteins, including BMPR1B and Krebs cycle enzymes, implicated in the pathogenesis of PAH (Joshi et al., 2020). While increased DNA hypermethylation leads to suppression of genes (*Sod2* and *Nos3*) encoding vasodilatory proteins and antioxidants in lungs of hypoxic mice, pulmonary artery SMCs of fawn‐hooded rats, and endothelial cells of fetal lamb with pulmonary hypertension (Archer et al., 2010; Joshi et al., 2020; Ke et al., 2018). Furthermore, DNA hypermethylation and downregulation of miR‐126 contributes to right heart failure in PAH (Potus et al., 2015), and play various roles in the regulation of genes during the development of atherosclerosis (Dong et al., 2002; Lacey et al., 2019). Because DNMT1 is the key maintenance methyltransferase, and DNMT3a and DNMT3b enzymes methylate unmethylated and hemimethylated CpG islands, we suggest increased DNMT3a and DNMT3b expression and activity potentially leads to *de novo* hypermethylation of genes in PAH patients. Furthermore, our results suggest higher *DNMT1* and lower *TET2* and *TET3* expression in Caucasian patients caused hypermethylation and downregulation of more protective genes genome‐wide, including those mentioned earlier, increasing severity of PAH.

In addition to increased expression of *DNMT* (1, 3a, and 3b), our results revealed PAH patients as compared with healthy controls had higher total DNMT activity, which is driven by metabolites of one‐carbon metabolism, the polyamine pathway, and the Krebs cycle (Puleston et al., 2017; Solary et al., 2014; Stover, 2009). Each of those pathways is reprogrammed in various cell types residing in the pulmonary artery and lungs of PAH patients and animal models (D'Alessandro et al., 2018). One‐carbon metabolism and polyamine pathway metabolites (s‐adenosylmethionine and spermine, respectively) are methyl‐group donors and regulators of DNA methylation (Amelio et al., 2014; Puleston et al., 2017) and are elevated in lungs/fibroblasts/SMCs of PAH patients (Joshi et al., 2020).

Along with higher expression and activity of DNMT, we found increased expression of *TET2* and *TET3* and total TET activity in PAH patients than control individuals. These findings are somewhat perplexing because lower expression of *TET2* in mononuclear cells was detected in >86% of inflammation‐associated PAH cases (Potus et al., 2020) and smooth muscle cells isolated from i‐PAH patients (Joshi et al., 2020). Although reasons for these differences in our results and previous studies are unclear, different source of genetic material (leukocytes vs. mononuclear cells/smooth muscle cells), methodologies (real‐time PCR vs. microarray) employed to measure gene expression, disease state, and race of the patients, presumably contributed to the observed differences. *TET2* expression levels were higher in Hispanic/African American than Caucasian patients who had more severe PAH. Therefore, we propose *TET2* and *TET3* expression and activity are increased to potentially antagonize actions of elevated DNMTs or vice versa in PAH patients.

Next, because loss‐of‐function mutation in TET2 and reduction of *TET2* expression is associated with cytokine production in PAH cases (Potus et al., 2020), we determined the expression of genes that encode cytokines in samples of Caucasian and Hispanic/African American patients. We found higher *CSF2* and *CCL5* levels significantly correlated with decreased *TET3*, but not with *TET2* and *DNMT1*, in Caucasian and Hispanic/African American patients. Increased *IL6* did not correlate with *DNMT1* and *TE2*/*TET3*, indicating *IL*‐*6* expression may not be directly regulated by altered DNA methylation status in Caucasian and Hispanic/African American patients. Interestingly, augmented *CD163* gene correlated with the expression of *DNMT1* but not with the expression of either *TETs*. Although DNMT1 is the key maintenance methyltransferase, down regulation miR‐124 by increased DNMT1 facilitates M1 alveolar macrophage polarization in acute lung injury (Wang et al., 2021). Furthermore, we observed significantly higher CD34^+^ cells and *CD34* gene expression in Caucasian than in Hispanic/African American patients. Since the expression of *CD34* and *CD163* were positively correlated, this potentially implies increased CD34^+^ cells convert to *CD163* gene expressing monocytes, and decreased *TET3* led to the upregulation of *CSF2* and *CCL5* in Caucasian and Hispanic/African American patients. Indeed, the reduction of *TET2* and *TET3* expression is responsible for hematologic disorders/malignancies and for increasing cytokine production (An et al., 2015; Solary et al., 2014). Inflammation caused by elevated cytokines, in perivascular region of the pulmonary artery, contributes to the remodeling of the pulmonary artery and increase pulmonary vascular resistance (El Kasmi et al., 2014). This is reflected by a positive correlation between PCWP and *TET2*/*TET3* expression. Therefore, it is not unreasonable to suggest that lower *TET2*/*TET3* expression contributed to the severity of PAH in Caucasian than in non‐Caucasian (Hispanic/African American) patients.

Although our findings clearly suggest differences in the expression and activity of epigenetic writers and erasers are potentially associated with the severity of PAH, these results should be cautiously interpreted as they could be confounded by the treatment for PAH. However, this is likely to be a minor point because there was no difference in expression of all genes, we examined, in Hispanic patients that were on treatment and one patient on no treatment. Furthermore, a small sample size in Caucasian and non‐Caucasian (Hispanic/African American) precludes us from affirmatively suggesting that differences in the epigenetics between the groups is a cause of the severity of PAH, the statistically significant differences between the groups certainly hints DNA methyltransferases and demethylases must be important to eliciting the severity of PAH in Caucasian group and this must be further studied. Nevertheless, DNMT and TET activity in circulating EVs could be further developed as diagnostic markers for determining the severity of PAH in different ethnic groups. EVs are cargos that transport second messengers, genetic information, and proteins (Blair et al., 2016; Sayner et al., 2019). They are emerging diagnostic tools. Therefore, further multicenter studies in more PAH patients with diverse ethnic backgrounds are needed to identify if circulating EVs are released from the lungs or leukocytes, and to correlate the activity of epigenetic enzymes in EVs with the severity of the disease for diagnostic purposes and determining the effectiveness of the therapy in PAH patients.

In summary, our findings support the hypothesis that increased DNMT‐catalyzed DNA methylation potentially contributes to the development and progression of PAH pathology in humans. Moreover, we have identified a potential association between expression and activity of increased DNMT1 and decreased TET2/TET3 and the excited inflammatory cytokine production and severity of PAH in Caucasian versus Hispanic/African American patients. Although some analyses suggest minorities may have a poor outcome in PAH, the role of ethnicity/race in PAH remains a controversial topic (Medrek & Sahay, 2018). Based on our findings, additional multicenter studies with more patients in different ethnic groups are warranted to confirm these findings and to develop new ethnicity/race‐based therapies for PAH.

## AUTHOR CONTRIBUTIONS

Catherine D’Addario: performed experiments, analyzed the data, and read the manuscript. Gregg M. Lanier: Collected samples and clinical data, and read the manuscript. Christina Jacob: performed experiments, analyzed the data, and read the manuscript. Natalie Bauer: designed extra‐vesicle particle isolation. Jenny L Hewes and Aritra Bhadra: performed EM and biochemical analysis of extra‐vesicle particles. Sachin A. Gupte: designed the study, analyzed the data, wrote the manuscript, and organized the publication.

## COMPETING INTERESTS

None.

## ETHICS APPROVAL AND CONSENT TO PARTICIPATE

This study was approved by the New York Medical College and Westchester Medical Center Institutional Review Board. A written consent was taken from each patient and control individuals prior to drawing blood.

## CONSENT OF PUBLICATION

All authors have read the manuscript and have given their consent of publication.
